# The Application of Handheld Near-Infrared Spectroscopy and Raman Spectroscopic Imaging for the Identification and Quality Control of Food Products

**DOI:** 10.3390/molecules28237891

**Published:** 2023-12-01

**Authors:** Hui Yan, Marina D. G. Neves, Barry M. Wise, Ingrid A. Moraes, Douglas F. Barbin, Heinz W. Siesler

**Affiliations:** 1 School of Biotechnology, Jiangsu University of Science and Technology, Zhenjiang 212100, China; yan_hui@just.edu.cn; 2 Department of Physical Chemistry, University Duisburg-Essen, 45117 Essen, Germany; marina.de.gea.n@gmail.com; 3 Eigenvector Research Inc., Manson, WA 98831, USA; bmw@eigenvector.com; 4 Department of Food Engineering and Technology, School of Food Engineering, University of Campinas, Campinas 13083-862, Brazil; i262845@dac.unicamp.br (I.A.M.); dfbarbin@unicamp.br (D.F.B.)

**Keywords:** handheld NIR spectroscopy, Raman spectroscopic imaging, extraction, clove, wolfberry, cocoa, powdered dairy, authentication

## Abstract

The following investigations describe the potential of handheld NIR spectroscopy and Raman imaging measurements for the identification and authentication of food products. On the one hand, during the last decade, handheld NIR spectroscopy has made the greatest progress among vibrational spectroscopic methods in terms of miniaturization and price/performance ratio, and on the other hand, the Raman spectroscopic imaging method can achieve the best lateral resolution when examining the heterogeneous composition of samples. The utilization of both methods is further enhanced via the combination with chemometric evaluation methods with respect to the detection, identification, and discrimination of illegal counterfeiting of food products. To demonstrate the solution to practical problems with these two spectroscopic techniques, the results of our recent investigations obtained for various industrial processes and customer-relevant product examples have been discussed in this article. Specifically, the monitoring of food extraction processes (e.g., ethanol extraction of clove and water extraction of wolfberry) and the identification of food quality (e.g., differentiation of cocoa nibs and cocoa beans) via handheld NIR spectroscopy, and the detection and quantification of adulterations in powdered dairy products via Raman imaging were outlined in some detail. Although the present work only demonstrates exemplary product and process examples, the applications provide a balanced overview of materials with different physical properties and manufacturing processes in order to be able to derive modified applications for other products or production processes.

## 1. Introduction

Although the IR and Raman spectroscopy techniques discussed here both fall under the heading of vibrational spectroscopy, they are based on different physical principles. In general, IR spectroscopy is divided into three categories of wavenumber ranges: near-infrared (12,500–4000 cm^−1^), mid-infrared (4000–400 cm^−1^), and far-infrared (400–10 cm^−1^) [1]. Mid-IR spectroscopy is based on the selective absorption of polychromatic radiation to excite fundamental vibrations of a variety of polar, chemical functionalities [2], while in NIR spectroscopy, overtone and combination vibrations are mainly excited by CH, OH, NH, SH, C=O, and C=C functionalities of the investigated material [2]. In the less frequently used far-infrared (or terahertz) spectroscopy, lattice vibrations and molecular rotations are mainly excited via the absorption of radiation. What all three techniques have in common is the requirement that the excited molecular movements lead to a change in the dipole moment (μ) of the functionalities involved. In Raman spectroscopy, which covers the wavenumber range of 4000–10 cm^−1^, on the other hand, monochromatic laser radiation of the visible or NIR range is used as a light source, and the incident radiation is inelastically scattered by the material under investigation with simultaneous excitation of fundamental vibrations of predominantly homonuclear functional groups (e.g., C=C, C=N, and aromatics). In contrast to IR spectroscopy, the excitation condition here is a change in the polarizability α (a measure of the deformation of the electron envelope of the vibrating functionality) during the excited oscillations [2].

The main reason for employing NIR spectroscopy is the ease of sample presentation. The disadvantage of broad and superimposed absorption bands can be readily overcome via the application of chemometric evaluation routines for qualitative and quantitative analyses [3].

Monochromator components used in NIR devices include grating micro spectrometers, Fourier-transform near-infrared (FT-NIR), microelectromechanical system (MEMS)- based FT-NIR, linear variable filters (LVFs), Hadamard transform systems, Fabry–Perot tunable filters, light-emitting diode (LED) arrays, and acousto-optic tunable filters (AOTFs) [4,5,6], resulting in the progressing miniaturization of the instrument.

Portable and handheld NIR spectrometers are easy to use and cheaper, making them a commercially feasible alternative for fast in-the-field investigations. As a result, they have become one of the most commonly used methods for non-destructive evaluation of a wide range of materials in different areas, such as agriculture [7,8,9,10], textiles [11], petrochemicals [12], polymer recycling [13], and pharmaceuticals [14,15].

For production practice, the price of instruments must be considered when promoting the use of the NIR technique. Based on the type of detector, the NIR spectrometers can be classified into two categories: array-detector instruments and single-detector instruments. For a single detector, the price is much lower; thus, to further reduce the hardware costs, new developments have focused on systems with single detectors. The DLP NIRscan Nano EVM (Dallas, TX, USA) is based on Texas Instruments’ digital micro-mirror device (DMD™) in combination with a single-element detector and covers the wavelength range from 900 nm to 1700 nm [16]. Another single-detector-based handheld NIR spectrometer, Si-Ware Systems (Cairo, Egypt), was introduced in the market. It is a MEMS FT-NIR spectrometer, which possesses the advantages of a wide wavelength range (1298–2606 nm) and easy data transmission between the device and a tablet [17].

The second important and powerful technique described in this paper for analyzing food is the Raman spectroscopic imaging method. Raman spectroscopic imaging is the instrumental combination of a microscope and a Raman spectrometer and can achieve sub-micrometer lateral resolution due to utilizing excitation wavelengths in the visible/near-infrared wavelength region. It has been based on the measurement of a complete spectrum per pixel, obtaining information on the spatial distribution of its constituents on the analyzed surface region of interest. In contrast, traditional spectroscopy only obtains information from the point of the sample where the radiation of the source was incident. The result of imaging spectroscopy is a three-dimensional arrangement (hypercube) of x and y spatial coordinates, with the third dimension containing the wavelength/wavenumber scale [18]. To obtain concentration distribution maps, it is necessary to unfold the three-dimensional arrangement into two dimensions. Raman spectroscopic imaging is an analytical method that provides the detection and quantification of compounds present on the analyzed surface. This technique can be employed for a wide range of materials, with food products being an important part [19,20]. Eksi-Kocak et al. investigated the detection of green pea adulteration in pistachio nut granules using Raman hyperspectral imaging [21]. Jiang et al. applied the hyperspectral imaging technique for the rapid detection and visualization of duck meat adulteration in beef [22]. Lohumi et al. studied the quantification of detection of multiple adulterants in wheat flour [19]. Song et al. applied Raman spectroscopic imaging for the detection of fish bones in fillets [23]. Different reviews on the technique of Raman spectroscopic imaging in food have been carried out in recent years, such as those by Hongbin et al. [24], Pathmanabana et al. [25], and Peterson et al. [26].

Many reference methods in routine food quality control are based on chromatographic techniques, such as HPLC, GC [27], and UPLC [28]. These techniques are highly sensitive in their analysis but require time-consuming sample preparation and the use of solvents. Near-infrared and Raman spectroscopy are techniques that considerably reduce the expenditure of time for sample preparation and the use of chemicals (such as solvents), making them interesting tools for efficient food analysis and quality control.

## 2. Applications of Handheld NIR Spectroscopy in Food Products

Food is one of the most important substances in everyday life; therefore, food processing, quality, and safety have always been an issue of great importance to people. With the ongoing development of new technologies, more and more new spectroscopic sensors with new applications are currently coming onto the market.

NIR and Raman spectroscopy are increasingly favored as analytical techniques as they not only contain valuable structural information about the material under investigation but, in combination with chemometric evaluation methods, also provide very rapid qualitative and quantitative results.

Recently, there have been many articles focusing on the use of handheld NIR spectrometers for the quality control of agricultural products and foodstuffs [29,30,31,32,33,34,35,36], pharmaceuticals [13,37,38,39], polymers [13,37,38], and forensic investigations [40,41]. Also, several recent reviews summarized the applications of this technique to the analysis of food, including milk and dairy products, meat, fish, fruits, and vegetables [16,42].

The aforementioned advancements in the miniaturization and affordability of handheld NIR spectrometers have made them an attractive tool for quantitative analysis and authentication to safeguard customers. Here, we present unpublished research results as the newest application examples that not only showcase the potential of handheld NIR spectrometers for food extraction analysis but also highlight their impact on quality control in daily life. In describing these application examples, we also discussed efforts to develop robust calibration models, including the use of external validation sample sets, replicate measurements of samples, variable selection algorithms, and spectral preprocessing algorithms.

### 2.1. Monitoring Food Extraction Processes

#### 2.1.1. Ethanol Extraction of Clove

Clove, the dried buds of the Myrtaceae plant *Eugenia caryophllata Thunb*, is one of the main food spices growing globally. Clove exhibits good effects as an antibacterial, antioxidant, and preservative [43,44,45], and is used as a natural and non-harmful preservative and flavoring additive in food products [45,46]. In addition, it possesses pharmacological effects as an analgesic, antipyretic, and anti-inflammatory formulation [46]. Furthermore, it encompasses antibacterial effects against typical food-related Gram-negative and Gram-positive bacteria. Its antimicrobial mechanism has been attributed to the ability of the phytochemical constituents to damage the cell walls and cell membranes of microorganisms, which results in cell death due to the loss of vital intracellular materials [47]. Another antimicrobial mechanism may also be due to cell membrane hyperpolarization [48].

The main active ingredients in clove are eugenol, beta-caryophyllene, and eugenyl acetate [49], in which eugenol is the main ingredient. It is a kind of phenolic that has a special fragrant smell, and is used against different pathogens [50]. Its pharmacological actions include antimicrobial, local anesthetic, pain relief, anti-inflammatory, and antitumor effects [51,52,53,54]. It can inhibit the production of proteases and amylase in *Bacillus cereus*, which induce cell lysis [55]. It was reported that eugenol blocked the synthesis of water-insoluble glucans in *Streptococcus mutans*, which inhibited the adherence of these bacteria on saliva-coated hydroxyapatite beads [56].

For the application as a medicine, the clove extract is more convenient than the original clove. Ethanol clove extract (ECE), containing eugenol, chlorogenic acid, monocaffeoylquinic acid, quercetin glucuronide, and gallic acid, is usually used to produce healthy tea [57,58]. Gonelimali et al. demonstrated that ECE exhibited a complete antimicrobial effect against the tested food-borne pathogens (*Escherichia coli*, *Vibrio parahaemolyticus*, *Pseudomonas aeruginosa*, *Salmonella enteritidis*, *Bacillus cereus*, *Staphylococcus aureus*, and *Candida albicans*) [48]. Its high antibacterial capacity is related to its phenolic content [59] by trapping intermediates of the Maillard reaction, scavenging free radicals, or by reacting with lipid radicals, which were constant, via the delocalization of unpaired electrons [60]. Furthermore, ECE exhibited comparable antioxidant activity to the synthetic antioxidant tertiary butylhydroquinone, and an ECE-prepared bio-composite film prolonged the storage stability of goat meat balls by controlling lipid oxidation and microbial growth [61].

In order to streamline the production of ECE, effective monitoring of the extraction process of ECE is crucial. For this monitoring procedure, NIR spectroscopy is obviously the most efficient technique. Thus, in what follows, the monitoring of the ECE extraction process via NIR spectroscopy has been discussed in some detail.

##### Materials and Methods

**Figure 1:**
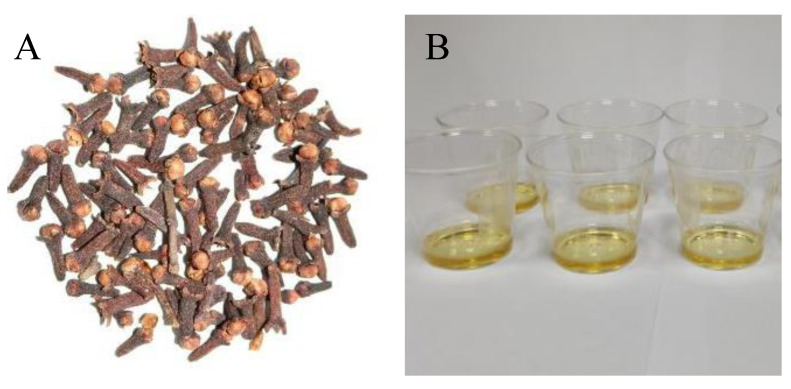
Photos of dried clove (**A**) and ECE solutions (**B**).

Clove was purchased from the Bozhou Traditional Chinese Medicine (TCM) market (Bozhou, Anhui, China). Dried clove (Figure 1A) was crushed and passed through a 40-mesh sieve to obtain clove powder, 200 g and 100 g of which were added to 600 mL and 1300 mL of ethanol at two solid–liquid ratios (g/mL) of 1:3 and 1:6.5, respectively, and were put in a shaker for extraction at 30 °C. At different extraction times, a 2 mL extraction of solution was collected. After centrifugation (8000 r/min) for 1 min, the supernatant (Figure 1B) was collected, weighed, and heated to 80 °C to volatilize ethanol until a constant weight was achieved. Finally, the residue was weighed, and the content of ECE was calculated.

A hierarchical method was employed to select the calibration and test sets [13,62]. The samples were arranged from small to large according to the reference values. Starting from the 2nd sample, one sample was selected every two intervals to form the test set, and the remaining samples formed a calibration set. Finally, 68 samples were collected as the calibration set and 34 samples as the prediction set, respectively.

**Figure 2:**
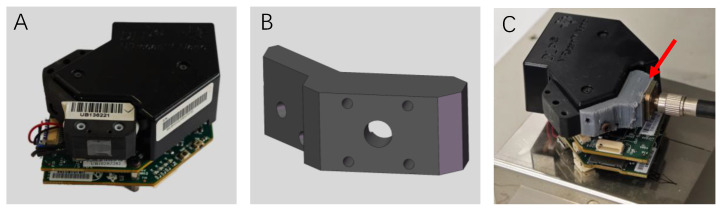
Schematic of the original spectrometer and the self-made modification. (**A**) Original DLP NIRscan Nano EVM. (**B**) Technical schematic of the 3D-printed adapter. (**C**) Modification of the DLP NIRscan Nano EVM with the 3D-printed adapter (red arrow).

**Figure 3:**
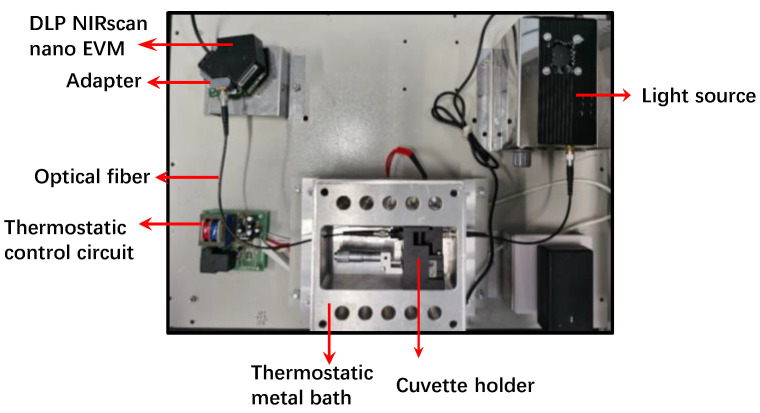
Photo of the individual modules in the light path of the experimental set-up.

For the measurement of NIR spectra, a low-cost, self-made NIR device was used. As shown in Figure 2, two illumination sources equipped in the original DLP NIRscan Nano EVM (Figure 2A) were dismantled, and an adapter (Figure 2B,C), which was customized using a 3D printer, was mounted to collect light from an optical fiber. The technical schematic of the 3D adapter (outside view) is shown in Figure 2B. As shown in Figure 3, the self-made set up was equipped with a 5 W H03 light source (Hangzhou Saiman Technology Co., Ltd., Hangzhou, China) and coupled to an optical fiber. The samples were measured in a 1 mm path length transmission flow cuvette in a 30 °C temperature-controlled box. A reference spectrum was recorded with an empty cuvette before the spectrum acquisition of samples. The spectral range was 900.1–1691.2 nm, and the spectral resolution was 7 nm. The spectrum of each sample was the average of 32 successive scans measured in 8.32 s. Each sample was measured three times, and the average of the three spectra was employed for further processing.

##### Results and Discussion

The reference values of ECE in the extraction solutions are summarized in Table 1. For the calibration set, the mean was 3.39% (*w*/*w*), and the standard deviation was 1.97% (*w*/*w*). The coefficient of variation (CV) derived from these parameters was 58.1%, indicating a large difference in content values.

**Figure 4:**
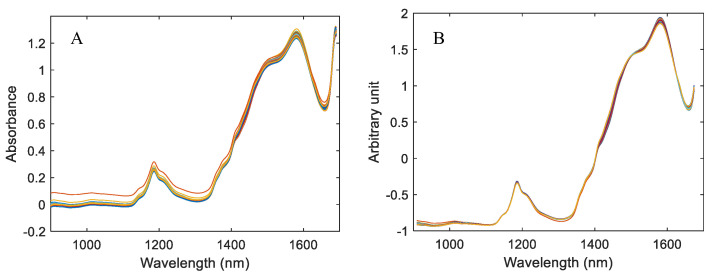
NIR spectra of the extraction solution of clove. (**A**) Raw spectra. (**B**) SNV pretreated spectra.

The raw NIR spectra are presented in Figure 4A and only exhibit a small ordinate shift. For further processing, the spectral range was limited to 909.3–1673.6 nm. Based on trial and error, the standard normal variate (SNV) pretreatment method proved the best calibration performance, and the SNV pretreated spectra are shown in Figure 4B.

Partial least squares (PLS) regression was applied to develop a calibration model. The calibration and validation statistics parameters were the R-squares (R^2^) and the root-mean-square error (RMSE), respectively, for the calibration (R_c_^2^, RMSEC), cross-validation (R_cv_^2^, RMSECV), and prediction (R_p_^2^, RMSEP) [63]. The residual predictive deviation (RPD_CV_) defined via the Std. Dev/RMSECV of the cross-validation set was also included to estimate how well the calibration model can predict the compositional data. Generally, a RPD_CV_ value greater than three can be considered as very good and suitable for prediction purposes [64].

**Figure 5:**
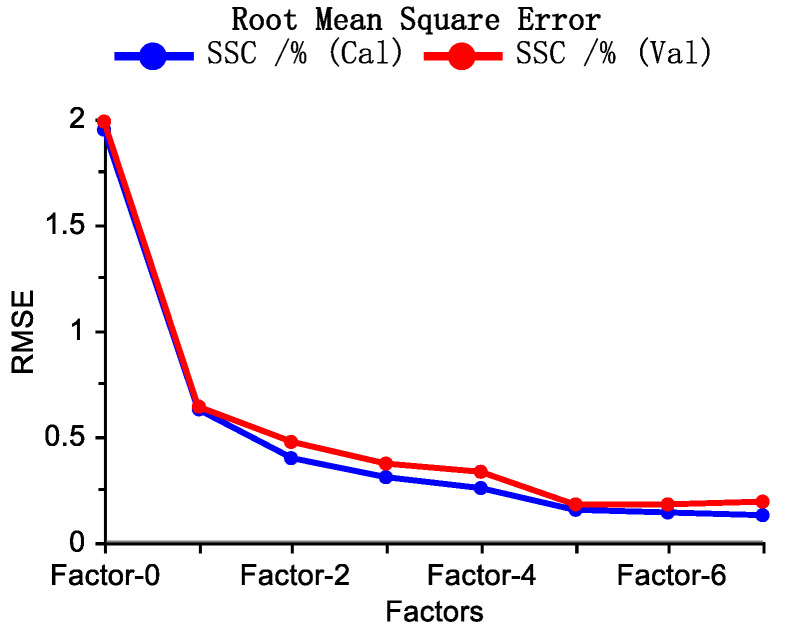
Factors versus RMSE plots for calibration (blue) and validation (red) for the selection of the optimal number of factors.

**Figure 6:**
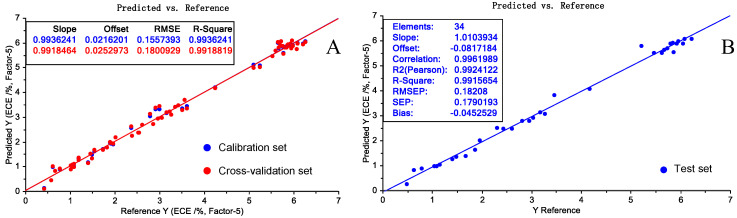
Reference versus predicted plots (including the most important performance parameters) for the calibration set (**A**) and the test set (**B**).

The number of optimal factors chosen for a calibration model exerts a significant impact on its prediction ability. According to Figure 5, the optimal number of factors was five. The reference versus predicted plots for the calibration and the test sets are presented in Figure 6A,B. The RMSEC and RMSECV values were 0.156% (*w*/*w*) and 0.180% (*w*/*w*), respectively, and their corresponding R_C_^2^ and R_CV_^2^ were 0.9936 and 0.9919, respectively. The low RMSE values and large R^2^ values demonstrate a high calibration performance. Furthermore, the RPD_CV_ was 10.94, which indicates that the calibration model can accurately determine the ECE over the whole concentration range. For the unknown test set, a high prediction performance was also achieved (Figure 6B), with RMSEP and R_P_^2^ values of 0.1821% (*w*/*w*) and 0.9916, respectively.

These data clearly demonstrate that the extraction process of ECE can be accurately monitored with the low-cost NIR device under investigation. It can also be applied for monitoring the extraction process of another material, which has been verified in the next application case.

#### 2.1.2. Water Extraction of Wolfberry

Wolfberry (*Lycium barbarum*) belongs to Solanaceae-defoliated shrubs, and its fruit is 1–2 cm long with a bright orange color [65]. It has been used as a herb in traditional Chinese, Korean, and Japanese medicine since at least the 3rd century AD [66]. Wolfberry has long been used to promote fertility and as a potent antiaging and antioxidant agent [67]. It was widely used as a foodstuff to nourish the liver and kidneys [68], and is frequently added to soups, hot pots, and herbal teas, and is also popularly soaked in wines alone or together with other ingredients of TCM to make functional wines [69].

The nutritional and functional properties of wolfberry originate from their components, including amino acids, polyphenols, flavonoids, carotenoids, polysaccharides, organic acids, and their derivatives [70,71,72], which encompass a lot of biological activities, such as antidiabetic, antiproliferative, preserving retinal function, and antioxidant activity [73]. It has also been found that the flavonoids from wolfberry protect the blood cells and mitochondria against oxidative damage [74].

The main components of soluble solids content (SSC) in wolfberry are polysaccharides, as well as some water-soluble alkaloids, flavonoids, and proteins. The SSC is a raw material for many health foods, especially in a number of health teas, containing some other extracts of ginseng, hawthorn, and chrysanthemum. When wolfberry is heated in water to boiling, the SSC is extracted into the water, and the SSC in water increases with extended heating time. In order to improve the yield of SSC and control the consumption of thermal energy, monitoring the SSC extraction process is extremely important. Thus, a fast detection method for the SSC via NIR spectroscopy in the wolfberry extraction process was investigated.

##### Materials and Methods

Wolfberry was purchased from Qing Yuantang Industrial Co., Ltd. (Luoyang, China). A total of 0.5 kg of wolfberry was added in 5 L of water at a solid–liquid ratio of 1:10 (*w*/*v*, kg/L), soaked for 1 h, heated to boiling, and then gently heated to maintain boiling for 180 min. Subsequently, it was filtrated with gauze, and the residue was added to 5 L to re-extract. In the two processes of extraction, 3 mL of extraction solution was collected in different time intervals; it was first filtrated with gauze while it was hot, and the filtered solutions were used for the measurement of NIR spectra and SSC determination. A hierarchical method was employed to select the calibration and test sets; finally, 40 and 20 samples were collected as the calibration set and the test set, respectively.

The reference values of SSC were determined using the heating to constant weight method, as described in the above section. In addition, the spectrum acquisition method with the customized NIR device was the same as for the ECE (Figure 3).

##### Results and Discussion

**Figure 7:**
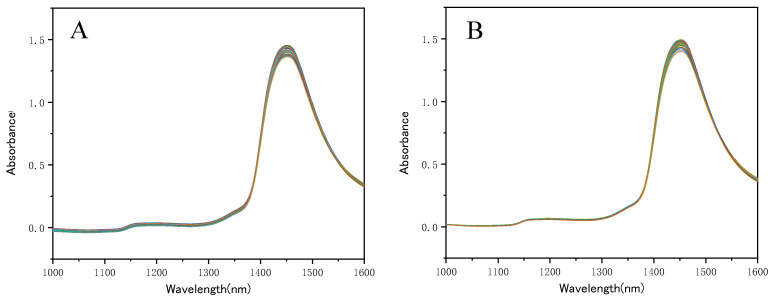
Raw NIR spectra of the SSC extraction solutions (**A**) and baseline corrected spectra (**B**).

The raw NIR spectra (1000–1600 nm) are shown in Figure 7A, in which the 2xν(OH) first overtone of water at 1450 nm is the strongest absorption peak. The baseline correction proved as the optimal spectrum pretreatment method, and the baseline corrected spectra are shown in Figure 7B.

**Figure 8:**
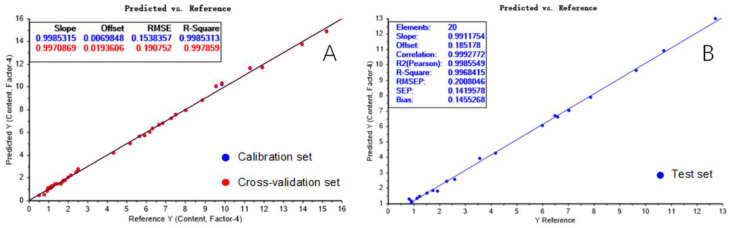
Reference versus predicted plots for the SSC values of the calibration set (**A**) and the test set (**B**).

The optimal number of factors chosen for the PLS calibration presented in Figure 8A was four. The RMSEC and RMSECV values were 0.154% (*w*/*w*) and 0.191% (*w*/*w*), respectively, and their corresponding R_C_^2^ and R_CV_^2^ values were 0.9985 and 0.9979, respectively. These parameter values demonstrate the high performance of the calibration model.

Twenty unknown samples were used as the test set, and the validation of the PLS model is represented by the reference versus predicted plot in Figure 8B. The RMSEP and R_P_^2^ values were 0.201% (*w*/*w*) and 0.9968, respectively, and demonstrated the robustness and high prediction capability of the model.

**Figure 9:**
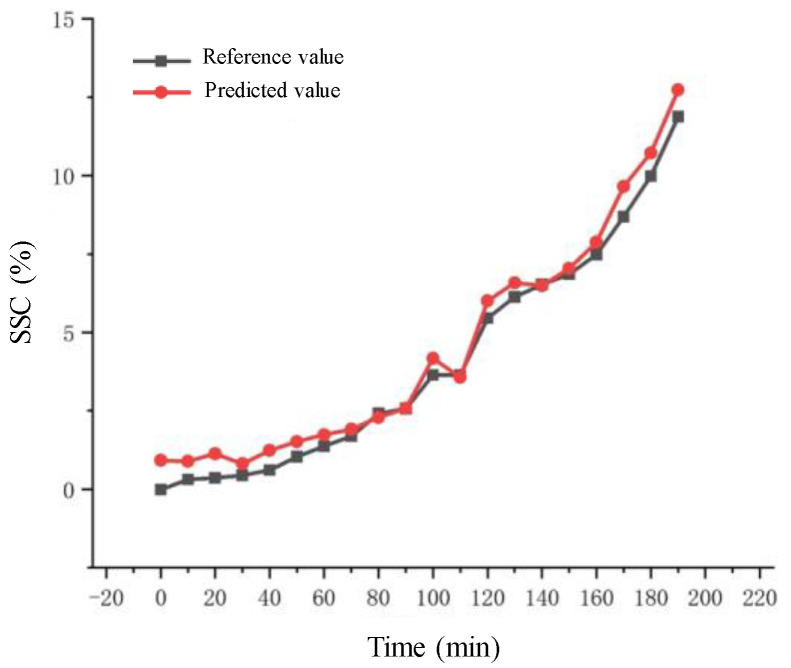
The SSC values determined using the two detection methods in the extraction process.

Figure 9 demonstrates the excellent agreement between the SSC values derived from the reference method and via NIR spectroscopy as a function of extraction time.

Based on these results, it is feasible that the NIR method for monitoring the wolfberry extraction process can be implemented into the industrial production process. Furthermore, it has broad application prospects, not only in the extraction process but also in fermentation, and in the food, bioenergy, and pharmaceutical sectors.

### 2.2. Differentiation of Cocoa Nibs and Cocoa Beans

Cocoa products have been the target of fraudulent processes in recent years, as they are widely used around the world. Many studies have been carried out with the aim of classifying and distinguishing different cocoa qualities [75], geographical origins [76], and predicting nutritional parameters, such as moisture, pH, acidity, protein, shell content, total phenolic compounds, and fat [77], using different techniques and physical–chemical tests [78]. Different types of fraud can be found in cocoa products through the addition of non-permitted ingredients [79] or higher content as declared on the label [80].

Nibs are a highly nutritious part of crushed cocoa beans. After harvesting, the cocoa beans are fermented, dried, and roasted. The shell is then removed to extract the nibs [81]. The presence of the shells in products from cocoa directly affects the final product quality and is undesirable. To increase profits, several products use whole cocoa beans or add cocoa shells to increase the final yield of the product. Due to the growing number of frauds, it is necessary to develop rapid techniques that allow the authentication of these products, such as handheld NIR spectroscopy. Benchtop NIR has been used for a while for cocoa laboratory analyses, but with the advancement of portable NIR spectroscopy, measurements can now be taken in the field, speeding up the quality control process [82].

#### 2.2.1. Materials and Methods

**Figure 10:**
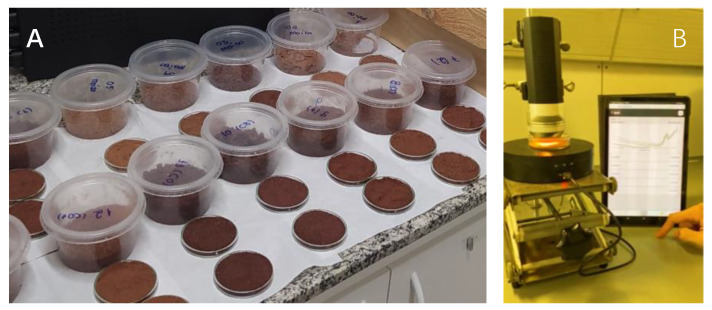
(**A**) Samples of cacao nibs and whole cacao nuts after grinding. (**B**) Sample presentation of a ground cocoa sample on the rotating dish device for spectra acquisition using the handheld NIR spectrometer.

A total of 35 nibs and 135 whole cocoa bean shell samples from four Brazilian states (Bahia, Pará, Amazonas, and Espírito Santo) provided by companies and cocoa farms were ground using a blender and analyzed using handheld NIR spectroscopy. The samples were put in a Petri dish and placed on a rotating accessory, measuring each sample three times (Figure 10B).

The spectra were acquired using a handheld LVF NIR device from trinamiX NIR Spectroscopy Solution, model SYS-IR-R-P (Ludwigshafen, Germany), with a spectral range from 1450 nm to 2450 nm, a spectral resolution of 15 nm (at 1500 nm), and a PbS line array detector. The data analyses were performed using MatLab R2023b and PLStoolbox 9.2 (Eigenvector, Manson, WA, USA). The data were pretreated via EMSC (extended multiplicative scatter correction) and mean centering.

#### 2.2.2. Results and Discussion

**Figure 11:**
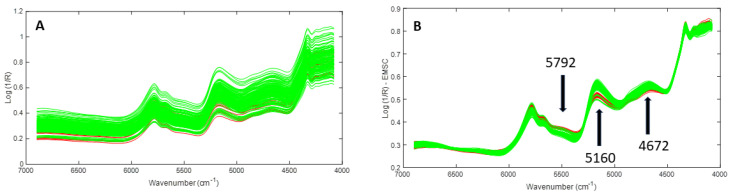
(**A**) Raw and (**B**) scatter-corrected NIR spectra of cocoa samples measured with the handheld device (green: whole cocoa beans; red: cocoa nibs).

In Figure 11A,B, the 170 raw and scatter-corrected cocoa NIR spectra, respectively, are shown. In green are the samples using the whole cocoa bean, and in red are the samples acquired from nibs. As emphasized by the arrows, three wavelength regions of spectral differences (5792, 5176, and 4672 cm^−1^) were identified. These bands were related to the 2ν(CH_2_) overtone vibrations of hydrocarbons, v(O-H) + δ(O-H) combination bands of polysaccharides and water residues, and ν(CH) + ν(C=O)+ δ(CH_2_) combination bands of lipids, respectively [83]. The NIR assignments discussed above, and other important bands, are summarized in Table 2.

**Figure 12:**
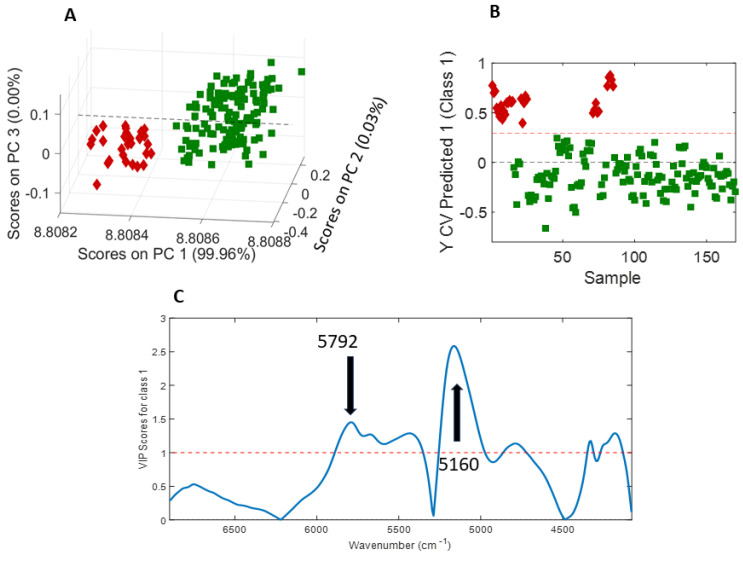
(**A**) Three-dimensional PCA score plot; (**B**) PLS-DA classification model; and (**C**) VIP scores for the 170 cocoa samples (green: cocoa beans; red: cocoa nibs).

Based on the application of using two different species of cocoa samples (nibs and whole beans), a PCA (principal component analysis) was developed with three PCs and is shown in Figure 12A. The third PC was added to represent the data in 3D space. In the 3D score plot of the PCA, it is possible to observe two clusters (red (nibs) and green (whole beans)). For the elaboration of a classification model, a PLS-DA (partial least squares-discriminant analysis) model was developed and is presented in Figure 12B. The dashed threshold line in red was used to distinguish between class 1 (red (cocoa nibs)) and class 2 (green (whole cocoa beans)). Class 1 samples were expected to be located above the threshold, and class 2 samples were expected to be located below the threshold. All the samples were classified correctly. The PLS-DA model only used one LV (latent variable) to distinguish between the two classes, achieving sensitivity and specificity values of 100%. The VIP (variable importance in projection) scores are shown in Figure 12C, and it can be seen that the bands at 5792 and 5160 cm^−1^ were critical for building the calibration model.

Despite showing no significant visual differences, cocoa powder samples vary with respect to their nutritional composition depending on whether only the nibs or the whole cocoa bean are used, especially in terms of fat and shell content. Thus, this simple example demonstrated the potential of handheld NIR spectroscopy in combination with the chemometric evaluation routines of PCA and PLS-DA to differentiate, in a rapid and efficient way, cocoa powder samples made from pure nibs and whole beans.

## 3. Application of Raman Spectroscopic Imaging

### 3.1. Identification and Quantification of Adulterations in Powdered Dairy Products

Milk is a globally consumed food, considered rich in nutrients and present in different diets and cultures [84]. Powdered milk stands out for its longevity, which makes it easy to transport and store, and can be used in times of shortage of fresh milk. In addition to the Chinese melamine scandal in 2008 [85], milk has been adulterated in various ways, for example, by adding water for dilution and adding constituents such as sucrose. Another source of adulteration can be the addition of nitrogenous products, such as urea and poor-quality whey [86].

Many different physicochemical analyses have been carried out to ensure the quality control of dairy products. This work aimed to use Raman imaging spectroscopy in combination with multivariate curve resolution-alternating least squares (MCR-ALS) [87,88] to simultaneously identify and quantify adulterations with sucrose, urea, and whey.

**Figure 13:**
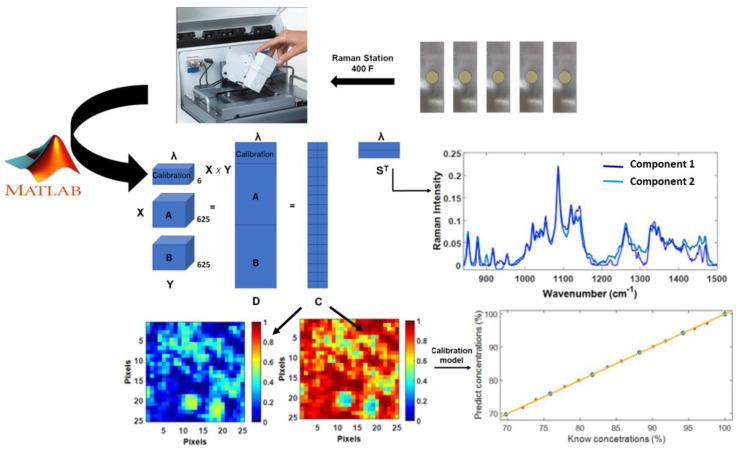
Experimental set-up and 3D hypercube structure of Raman spectroscopic imaging data, followed by 2D matrix unfolding. The MCR-ALS algorithm decomposes the matrix (**D**) into a spectral (**S**) and a concentration (**C**) profile. This allows to recover the spectral profiles, the calculation of distribution maps, and the quantification of the components.

Imaging spectroscopy is based on the measurement of a complete spectrum per sample surface unit (pixel), obtaining information about the spatial distribution of its constituents on the analyzed surface area. The result of imaging spectroscopy is a three-dimensional arrangement of *x*/*y* spatial coordinates, with the wavenumber/Raman shifts as the third dimension. To obtain the distribution maps, it is necessary to unfold the three-dimensional arrangement into two dimensions and apply the MCR-ALS algorithm (Figure 13).

#### 3.1.1. Materials and Methods

The samples were analyzed using a Raman station 400F (Perkin Elmer, Waltham, MA, USA), with an excitation laser of 785 nm, a pixel size of 50 μm, a spectral range of 3200–200 cm^−1^, and a spectral resolution of 2 cm^−1^. In total, 625 spectra (25 × 25 pixels) were acquired over an area of 2.4 mm^2^. The laser was used at its maximum power 250 mW (at source), with an exposure time of 1s, and the exposure was repeated five times with subsequent averaging.

The data were processed using Matlab R2023b^®^ software and the MCR-ALS 2.0 toolbox (2018)^®^. The spikes due to ambient light were removed manually using the algorithm gpsabin, and the SVD (singular value decomposition) [89] was used to select the number of components. The initial estimates of the spectral profiles were generated using the SIMPLISMA algorithm (simple-to-use interactive self-modeling mixture analysis) [90]. Non-negativity in both the concentration and spectral profiles and correlation in the concentration matrix were used as constraints.

Different sets of physical mixtures were prepared containing milk powder and the following adulterants: whey, sucrose, and urea. To ensure better homogeneity, the products were ground and mixed using a vortex machine. For the calibration, the milk powder was mixed with the adulterants, one at a time, in a range of 0–30% (*w*/*w*) at 5% intervals, resulting in seven samples. The seven calibration images were averaged, resulting in seven individual spectra for each adulterant. The data were processed using the augmented matrix technique, where all the images were concatenated one below the other, and a predictive model was built for each adulterant. For the prediction set, 27 new samples containing a mixture of powdered milk and adulterants were prepared.

The samples were prepared with whole milk powder of the commercial brand Piracanjuba, lot 1147/1, and the adulterants used were sucrose (C_12_H_22_O_11_) and urea (CH_4_N_2_O) from Synth lot 146789 and Sigma lot 68H01161, respectively. The whey used in the experiment was characterized as a poor-quality product and was donated by ITAL—Institute of Food Technology (Campinas, Brazil).

Four prediction samples were discussed in detail in this manuscript (Table 3). Further samples can be found in Neves et al. [91].

#### 3.1.2. Results and Discussion

**Figure 14:**
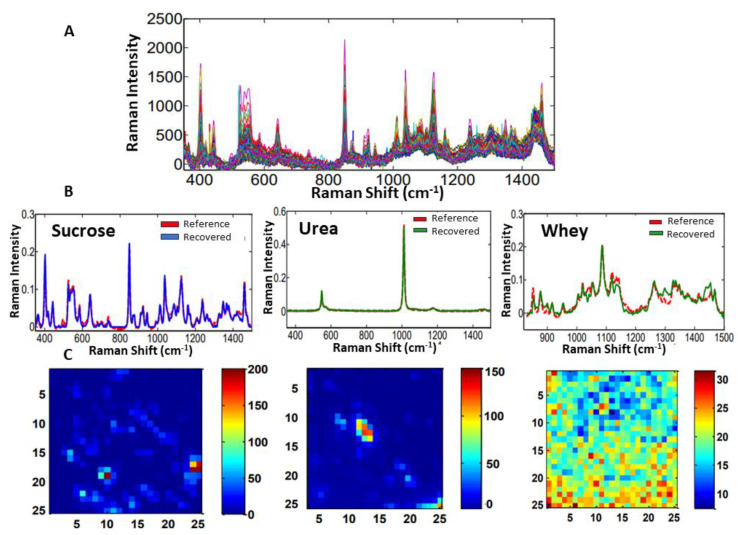
(**A**) Raman spectra for sample #1, (**B**) reference and recovered spectral profiles, and (**C**) distribution maps of sucrose, urea, and whey used as adulterants.

From the MCR-ALS technique, it is possible to recover the spectral profile and calculate the distribution maps for each sample and for each component of the system (adulterants and milk). The spectra recovered for each component were compared in terms of similarity to the reference spectra, which were obtained by measuring the pure adulterants. In this way, the greater the overlap between the spectra, the more similar they are to each other. In case of sample #1, the spectra recovered for sucrose and urea almost completely overlapped with the reference spectra (Figure 14). The whey spectrum showed a larger difference, mainly because it is very similar to the milk matrix and therefore has some characteristics of that component. The maps represent the distribution of the components in each pixel on the surface of the mapped area. The red color indicates a higher concentration, while the blue color denotes a lower concentration. Figure 14 shows the Raman spectra, recovered spectral profiles, and distribution maps of sucrose, urea, and whey used as adulterants for sample #1.

**Figure 15:**
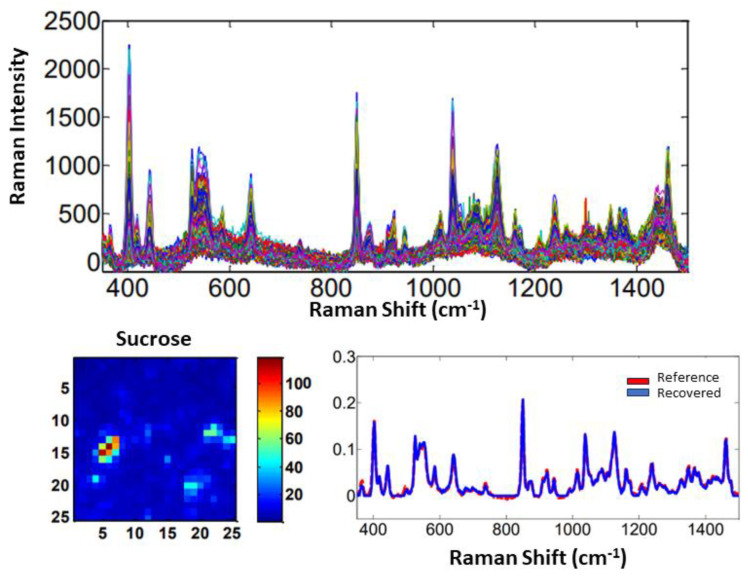
Raman spectra for sample #2, distribution map, and reference and recovered spectral profiles of sucrose as an adulterant.

**Figure 16:**
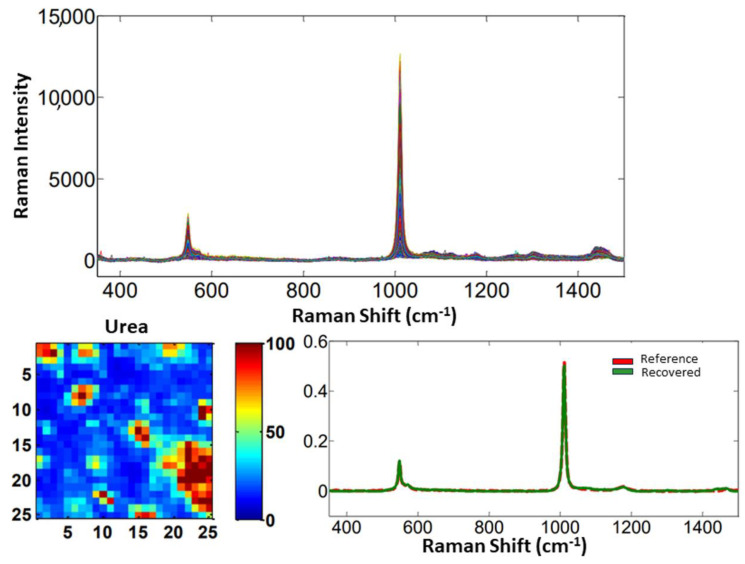
Raman spectra for sample #3, distribution map, and reference and recovered spectral profiles of urea as an adulterant.

**Figure 17:**
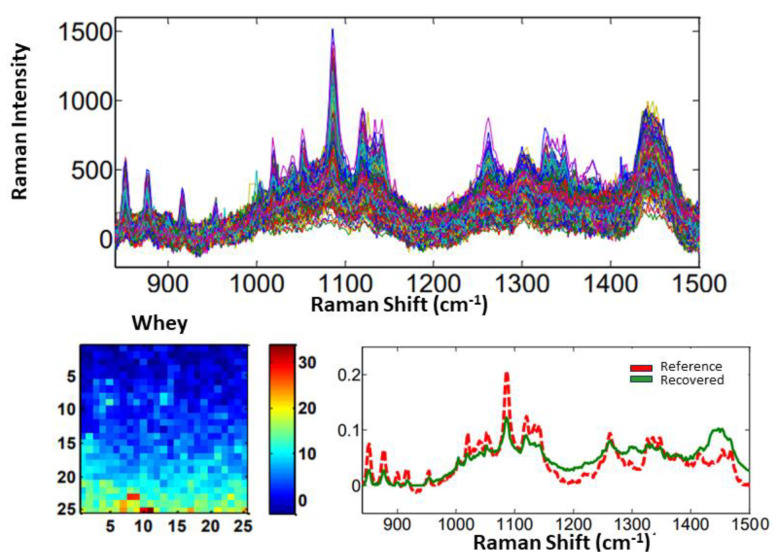
Raman spectra for sample #4, distribution map, and reference and recovered spectral profiles of whey as an adulterant.

For the samples #2, #3, and #4, which only contained one adulterant, the Raman spectra, reference and recovered spectral profiles, and distribution maps are shown for sucrose, urea, and whey, respectively, in Figure 15, Figure 16 and Figure 17.

As for sample #1, the recovered spectra with the greatest similarities to the reference spectra are urea and sucrose. From the concentration profile and recovered spectra, the distribution maps were calculated. Then, from each distribution map, the mean concentration values in % (*w*/*w*) were calculated and presented with their absolute errors in Table 4. The number of factors selected for samples #1/#2/#3/#4 were 4/2/2/3, respectively.

The proposed methodology was suitable for the detection and quantification of sucrose, urea, and whey as adulterants in powder milk samples, and the absolute average errors were below 2% (*w*/*w*). Thus, with the combination of Raman imaging spectroscopy and MCR-ALS, it is possible to quantify adulterants in the presence of interferents not present in the calibration, known as second-order advantage. The augmented matrix approach requires a shorter treatment time but needs an equal number of factors for all samples used. If the samples are very different from each other, the strategy of using individual matrices should be investigated.

## 4. Conclusions

The NIR test results have shown that the ECE (clove) and SSC (wolfberry) extraction processes can be monitored with sufficient accuracy using the low-cost NIR spectrometer, and that the device can also be integrated into the industrial production process. Furthermore, it has broad application prospects not only in extraction processes but also in fermentation, and in the food, bioenergy, and pharmaceutical sectors.

The classification models demonstrated for cocoa samples proved promising when using NIR spectroscopy in combination with PLS-DA. However, in the next stage, additional test sets for external validation should be evaluated. Furthermore, the development of calibration models for other parameters that affect the final quality of the product, such as the cultivation method (organic/conventional) and the geographical origin, should be considered.

The simultaneous quantification of three adulterants in milk powder matrices using MCR-ALS and Raman spectroscopic imaging showed satisfactory results with errors of less than 2% (*w*/*w*). The strategy of using an augmented matrix to process the data reduces the time needed to analyze and create the models but has the limitation of using the same number of factors for all the samples analyzed. Future studies should compare the augmented matrix method and individual approaches for the development of prediction models. We also suggest to investigate the feasibility of detecting lower concentrations of adulterants.

Both techniques, handheld NIR spectroscopy and Raman spectroscopic imaging, have proven their potential with respect to the application examples discussed in the present work in combination with the respective chemometric evaluation methods. The examples reported include samples under different morphological aspects, including powder and liquid samples, demonstrating the wide range of applications for NIR and Raman spectroscopy. Especially, the possibility of the on-site and in-process use of NIR spectroscopy and the high lateral resolution of Raman spectroscopic imaging make both techniques indispensable analytical tools in the context of quality and production control for the food industry.

## Figures and Tables

**Table 1:** The normal distribution-based descriptive statistical analysis for the ECE of the extraction solution.

Sample Set	Mean % (*w*/*w*)	Max. % (*w*/*w*)	Min. % (*w*/*w*)	Range % (*w*/*w*)	Std. % (*w*/*w*)	CV (%)
Calibration	3.39	6.27	0.42	5.85	1.97	58.1
Test	3.46	6.27	0.49	5.78	2.05	59.2

**Table 2:** Band assignments of the NIR spectra of powder samples produced from whole cocoa beans and cocoa nibs.

Wavenumber (cm^−1^)	Functional Group	Material Type
5792	C-H (2ν(CH_2_) overtone)	Hydrocarbons and aliphatic
5672	C-H (2ν(CH_2_) overtone)	Hydrocarbons and aliphatic
5176	v(OH) + δ(OH) combination	Polysaccharides and water
4880	N-H in-plane bending + ν(C-N) combination	Amides/proteins
4672	ν(CH) + ν(C=O) + δ(CH_2_) combination	Lipids
4328	C-H (3δ(CH_2_) overtone)	Lipids
4264	C-H (2ν(CH_2_) + δ(CH_2_) combination)	Hydrocarbons and aliphatic

**Table 3:** Percentage (*w*/*w*) concentration values used for test sample preparation.

Sample	Sucrose (%)	Urea (%)	Whey (%)	Milk (%)
#1	10	5	15	70
#2	10	-	-	90
#3	-	30	-	70
#4	-	-	5	95

**Table 4:** Average concentration values (% *w*/*w*) and the absolute errors (% *w*/*w*) for sucrose, urea, and whey used as adulterants in four different prediction samples.

Sample	Sucrose		Urea		Whey	
	Pred	Abs Error	Pred	Abs Error	Pred	Abs Error
#1	9.1	−0.9	6.3	1.3	17.8	2.8
#2	9.2	−0.8	-	-	-	-
#3	-	-	30.9	0.9	-	-
#4	-	-	-	-	4.5	−0.5
Average		0.85		1.10		1.65

Pred = predicted; Abs = absolute.

## Data Availability

Data are contained within the article.
